# Comparison of the Efficacy and Safety Profiles of Different P2Y12 Inhibitors in Patients With ST-Segment Elevation Myocardial Infarction in the COVID-19 Era

**DOI:** 10.7759/cureus.43829

**Published:** 2023-08-20

**Authors:** Ali̇ N Kaya, Mürsel Şahin

**Affiliations:** 1 Cardiology, Hakkari State Hospital, Hakkari, TUR; 2 Cardiology, Karadeniz Technical University Medical School, Trabzon, TUR

**Keywords:** thrombosis, mortality, covid-19, antiplatelet agents, acute coronary syndrome

## Abstract

Background/aim: Coronavirus Disease 2019 (COVID-19) is characterized by an increased risk of thrombotic and hemorrhagic events resulting from endothelial dysfunction. In patients with ST-elevation myocardial infarction (STEMI), the dual antiplatelet therapy used to reduce mortality may increase the risk of bleeding. The study aimed to compare the efficacy and safety profiles of P2Y12 inhibitors used during the COVID-19 era.

Materials and methods: Three hundred and ninety patients who underwent primary percutaneous intervention for STEMI between January 1, 2020, and December 31, 2021, were included in this study, retrospectively. The patients were divided into groups according to their COVID-19 history and all-cause mortality, cardiac mortality, stent thrombosis, and bleeding complications during hospitalization and at one-year follow-up were compared.

Results: The mean age of the patients was 64.3 years and the mean follow-up period was 10.2 months; 80% of the patients were male and 44.6% had a history of COVID-19 infection. The in-hospital mortality rate was 11.3%. Cardiac mortality was significantly higher in the clopidogrel group compared to the other groups, regardless of COVID-19 history (21.9% in the clopidogrel group, 1.6% in the prasugrel group, and 6.7% in the ticagrelor group (p<0.001)). There was no significant difference between the groups in terms of bleeding complications and relation to COVID-19.

Conclusion: In STEMI patients treated with different P2Y12 inhibitors, there was no significant difference in mortality, bleeding, stroke, and thrombotic complications, regardless of the presence or absence of COVID-19 infection.

## Introduction

Coronary artery disease (CAD) is prevalent worldwide and is a major cause of mortality. During the COVID-19 pandemic, individuals with cardiovascular risk factors experienced a higher incidence of both mortality and morbidity. Additionally, there has been a notable rise in cases of acute coronary syndrome (ACS) during this period, which is likely linked to the impact of the virus [1,2].

In patients with ST-elevation myocardial infarction (STEMI), dual antiplatelet therapy with an appropriate P2Y12 inhibitor should be administered in addition to acetylsalicylic acid (ASA) therapy after percutaneous coronary intervention (PCI) as recommended by the guidelines. The efficacy and safety profiles of P2Y12 inhibitors should be evaluated and maintenance treatment with prasugrel or ticagrelor, which are potent in terms of efficacy, should be provided in patients if there is no contraindication [3].

Signs of COVID-19 infection range from mild respiratory symptoms to severe cardiovascular and pulmonary complications. Although the disease mostly affects the respiratory tract and patients usually die from respiratory failure, cardiovascular complications are known to be associated with significant mortality. The COVID-19 virus affects multiple organs through various pathways, and one of the complications observed in patients is coronary artery thrombosis. This occurs due to the disruption of plaque stabilization caused by increased inflammation [4,5,6]. Several studies have indicated that individuals with STEMI who are also diagnosed with COVID-19 are more likely to experience stent thrombosis as a result of an elevated thrombus burden [7,8,9].

The study aimed to evaluate the effects of different P2Y12 inhibitors on stent thrombosis and bleeding in patients with STEMI during the COVID-19 pandemic. The findings of this study will contribute to the adjustment of medical treatment of these patients during the ongoing COVID-19 pandemic and similar pandemics that may occur in the future.

## Materials and methods

This study included all patients admitted with a diagnosis of STEMI during the COVID-19 pandemic between January 1, 2020, and December 31, 2021, retrospectively. Demographic characteristics; clinical risk factors, including hypertension, diabetes, atrial fibrillation, heart failure, history of previous cardiac events, cerebrovascular disease, peripheral arterial disease, chronic kidney disease, smoking history, and COVID-19 infection; as well as patients’ medical treatment and biochemical parameters at discharge were recorded. Patients who had a positive COVID-19 PCR test at the time of hospital admission or who had COVID-19 infection in the last six months and during the follow-up period were considered to be in the COVID-19-positive group. Patients with uncertain COVID-19 information and insufficient follow-up data were not included in the study.

All patients underwent primary percutaneous intervention as recommended by the guidelines. Again, all patients received medical treatment as recommended by the guidelines. The dual antiplatelet treatment option was left to the discretion of the physician. Clinical outcomes, such as major or minor post-procedural bleeding, stent thrombosis, cardiogenic shock, and death, were recorded during the hospitalization. Major bleeding was described as a reduction of 5 g/dL or more in the hemoglobin levels, a decrease of 15% or more in the hematocrit, or intracranial bleeding. Minor bleeding was characterized as a reduction in hemoglobin levels of 3-5 g/dL or a decrease in hematocrit levels of 10-15%, as well as bleeding in the gastrointestinal or urinary tract.

Hospital admissions with ACS, need for revascularization, cardiac or all-cause mortality, stroke, hospitalization with heart failure, and major/minor bleeding outcomes were recorded during the one-year follow-up using the hospital records or telephone visits.

This study is approved by the institutional ethics committee (Karadeniz Technical University, Ethics Committee, 25/07/2022, No:1) and complies with Helsinki Declaration.

Statistical analysis

Statistical Package for the Social Sciences version 23.0 (IBM Corp., Armonk, New York, 2017) program was used to analyze the data obtained from the study. The Kolmogorov-Smirnov test was used to assess conformity with normal distribution. The Kruskal-Wallis test was utilized to compare non-normally distributed data across groups consisting of three or more, and multiple comparisons were conducted using Dunn’s test. In paired groups, the independent samples T-test was used to compare normally distributed data, and the Mann-Whitney U Test was used to compare non-normally distributed data. Pearson chi-square test, Yates correction, and Fisher’s exact test were used for comparisons according to categorical data, and multiple comparisons were made using the Bonferroni correction. The Kaplan- Meier curve analyzed deaths during the one-year follow-up period. The results of the analyses were presented as frequency (percentage) for categorical variables and mean±standard deviation and median (minimum-maximum) for quantitative variables. The significance level was set at p<0.050.

## Results

A total of 390 patients were included in the study. The mean age of the patients was 64±12 years and the mean follow-up period was 10.2±4 months; 80% of the patient group was male. The characteristics of the study population are given in Table 1. 

**Table 1:** Baseline characteristics of patients PCI, percutaneous coronary intervention; CABG, coronary artery bypass graft; COPD, chronic obstructive pulmonary disease; DAPT, dual antiplatelet treatment; ACE, angiotensin-converting enzyme; LAD, left anterior descending artery; MACE, major adverse cardiac event; RCA, right coronary artery; IMA, intermediate artery; Cx, circumflex artery

Female gender n, (%)	78 (20)
Age	33-96(64)
Hypertension n, (%)	261 (66.9)
Diabetes mellitus n, (%)	128 (32.8)
Atrial fibrillation n, (%)	30 (7.7)
Heart failure n, (%)	23 (5.9)
Previous PCI history n, (%)	82 (21)
Previous CABG history n, (%)	14 (3.6)
Chronic kidney disease n, (%)	88 (22.6)
Asthma/COPD n, (%)	25 (6.4)
Smoking n, (%)	213 (54.6)
COVID-19 positive	174 (44.6)
Acetylsalicylic acid n, (%)	380 (97.4)
P2Y12 inhibitor	
Ticagrelor n, (%)	105 (26.9)
Clopidogrel n, (%)	160 (41)
Prasugrel n, (%)	125 (32.1)
Switch on admission n, (%)	10 (2.6)
DAPT duration (months)	10.01±4.45
Warfarin n, (%)	5 (26.3)
Direct oral anticoagulant n, (%)	14 (73.7)
Beta blocker n, (%)	358 (91.8)
ACE inhibitor n, (%)	275 (70.5)
Statin n, (%)	390 (100)
Hemoglobin (g/dL)	14.19±2.03
Platelet count (×10^3^/μL)	239.5 (53-794)
Creatinine (mg/dL)	1.11 ±0.68
Glomerular filtration rate (mg/dL)	79 (7-199)
Troponin (ng/dL)	76 (3-10000)
Low-density lipoprotein (mg/dL)	120 (25-276)
Infarct related vessel	
LAD n, (%)	160(41)
CX n, (%)	55 (14.1)
RCA n, (%)	163 (41.8)
IMA n, (%)	3 (0.8)
Saphen graft n, (%)	9 (2.3)
Non-culprit lesion n, (%)	40 (156)
Multivessel revascularization n, (%)	106 (67.9)
Total stent length (mm)	35.5 (9-129)
Ejection fraction (%)	42.8±9
Valve disease n, (%)	145 (37.2)
MACE during hospitalization	
Minor/major bleeding n, (%)	10 (2.6)
Stent thrombosis n, (%)	4 (1)
Cardiovascular mortality n, (%)	44 (11.3)
One-year follow-up MACE	
Acute coronary syndrome n, (%)	48 (13.9)
Stent thrombosis n, (%)	3 (0.9)
Cardiovascular mortality n, (%)	17 (4.9)
Stroke n, (%)	3 (0.9)
Hospitalization for heart failure n, (%)	52(15)
Minor/major bleeding n, (%)	6 (1.7)

ASA use was 97.4%. The mean duration of dual antiplatelet treatment (DAPT) in the patient group was 10 months. The patients were categorized based on the type of P2Y12 inhibitors and a comparison was made between the variables (Table 2).

**Table 2:** Comparison of variables according to P2Y12 inhibitors Pearson chi-square test; frequency (percentage); a-c: There is no difference between groups with the same letter. Mean±standard deviation; median (minimum-maximum) if a quantitative variable followed a normal distribution, the arithmetic mean was utilized, whereas for those that did not follow a normal distribution, the median was used. PCI, percutaneous coronary intervention; ACE, angiotensin-converting enzyme; ARB, Angiotensin receptor blockers

	P2Y12 Inhibitor			p
	Ticagrelor (n=105)	Clopidogrel (n=160)	Prasugrel (n=125)	
Female gender n, (%)	14 (13.3)^a^	50 (31.3)^b^	14 (11.2)^a^	<0.001
Age (year)	59 (38-77)^a^	76 (40-96)^b^	57 (33-76)^c^	<0.001
Hypertension n, (%)	69 (65.7)^ab^	121 (75.6)^b^	71 (56.8)^a^	0.003
Diabetes mellitus n, (%)	36 (34.3)	57 (35.6)	35 (28)	0.37
Previous PCI n, (%)	16 (15.2)^a^	49 (30.6) ^b^	17 (13.6)^a^	0.001
Chronic kidney disease n, (%)	17 (16.2)^a^	62 (38.8)^b^	9 (7.2)^a^	<0.001
Smoking n, (%)	62 (59)^a^	64 (40)^b^	87 (69.6)^a^	<0.001
COVID-19 history n, (%)	51 (48.6)^ab^	81 (50.6)^b^	42 (33.6)^a^	0.010
ACE/ARB inhibitor n, (%)	81 (77.1)^a^	87 (54.4)^b^	107 (85.6)^a^	<0.001
Hemoglobin (g/dL)	14.6±1.5^b^	13.2±2.0^a^	15.0±1.8^b^	<0.001
Platelet (×10^3^/μL)	244 (82-794)^ab^	220.5 (53-662)^a^	251 (83-545)^b^	0.005
Creatinine (mg/dL)	1.06±0.62^b^	1.3±0.86^a^	0.93±0.31^b^	<0.001
Glomerular filtration rate (mg/dL)	80.5±23.4^a^	60.2±24.1^b^	87.1±16.4^c^	<0.001
Troponin (ng/dL)	58 (3-5689)^b^	127.5 (3.8-10000)^a^	58 (3-8613)^b^	<0.001
Low-density lipoprotein (mg/dL)	124 (26-223)^b^	109 (25-226)^a^	124 (52-276)^b^	0.001
Ejection fraction (%±SD)	44.7±8.3^b^	39.9±10.1^a^	44.9±8.8^b^	<0.001
Valve disease n, (%)	34 (32.4)^a^	92 (57.5)^b^	19 (15.2)^c^	<0.001
Cardiogenic shock n, (%)	11 (10.5)^a^	36 (22.5)^b^	4 (3.2)^a^	<0.001
Cardiovascular mortality n, (%)	7 (6.7)^a^	35 (21.9)^b^	2 (1.6)^a^	<0.001
Hospitalization for heart failure n, (%)	8 (8.2)^a^	40 (32)^b^	4 (3.3)^a^	<0.001

Cardiovascular mortality rates during hospitalization were statistically significant between the groups (21.9% in the clopidogrel group, 1.6% in the prasugrel group, and 6.7% in the ticagrelor group (p<0.001)). Stent thrombosis was observed in only four patients (1%) during hospitalization. Of these patients, three were initially given a loading dose of clopidogrel and one was given a loading dose of ticagrelor, and the switch to prasugrel was performed during the follow-up. No major bleeding was observed during hospitalization, and the minor bleeding rate was 2.6%. During the hospital stay, a total of ten patients (three patients using clopidogrel, three using ticagrelor, and four using prasugrel) had minor bleeding, such as hematuria and lower gastrointestinal bleeding, that did not significantly decrease the hemoglobin value.

At the one-year follow-up, the rate of hospital admission with ACS was 13.9%, the rate of revascularization requirement was 12.4%, the rate of stent thrombosis was 0.9%, the rate of cardiac mortality was 4.9%, the rate of all-cause mortality was 2.9%, the rate of stroke was 0.9%, the rate of hospitalization with NYHA class 4 heart failure symptoms was 15%, and the rate of minor/major bleeding was 1.7%. Stent thrombosis was observed in a total of three patients (0.9%), two patients on clopidogrel and one on ticagrelor. Stroke/transient ischemic attack was observed in two patients on clopidogrel and one patient on ticagrelor (0.9%). No major bleeding was observed in the patients, and minor bleeding was observed in a total of six patients, including three patients using clopidogrel, one patient using ticagrelor, and two using prasugrel.

The baseline characteristics of patients according to the history of COVID-19 infection were compared. No statistically significant difference was found between the other variables except hypertension (Table 3).

**Table 3:** Comparison of variables according to COVID-19 history Pearson chi-square test; frequency (percentage); a-c: There is no difference between groups with the same letter. Mean±standard deviation; median (minimum-maximum) if a quantitative variable followed a normal distribution, the arithmetic mean was utilized, whereas for those that did not follow a normal distribution, the median was used. MACE, major adverse cardiac event

	COVID-19 history		Total	p
	No (n=216)	Yes (n=174)		
Age	62 (33-93)	64 (36-96)		0.611
Female gender n, (%)	46 (21.3)	32 (18.4)	78 (20)	0.476
Hypertension n, (%)	132 (61.1)	129 (74.1)	261 (66.9)	0.007
Diabetes mellitus n, (%)	66 (30.6)	62 (35.6)	128 (32.8)	0.289
Smoking n, (%)	119 (55.1)	94 (54)	213 (54.6)	0.833
P2Y12 inhibitor				
Ticagrelor n, (%)	54 (25)^a^	51 (29.3)^a^	105 (26.9)	
Clopidogrel n, (%)	79 (36.6)^a^	81 (46.6)^b^	160 (41)	0.010
Prasugrel n, (%)	83 (38.4)^a^	42 (24.1)^b^	125 (32.1)	
Glomerular filtration rate (mg/dL)	82 (7-118)	76 (7-199)		0.444
Low-density lipoprotein (mg/dL)	121 (25-226)	116.5 (26-276)		0.255
MACE during hospitalization				
Minor/major bleeding n, (%)	6 (2.8)	4 (2.3)	10 (2.6)	-
Stent thrombosis n, (%)	1 (0.5)	3 (1.7)	4 (1)	-
Cardiovascular mortality n, (%)	22 (10.2)	22 (12.6)	44 (11.3)	0.362
One-year follow-up MACE				
Cardiovascular mortality n, (%)	7 (3.6)	10 (6.6)	17 (4.9)	0.463
Stent thrombosis n, (%)	1 (0.5)	2 (1.3)	3 (0.9)	-
Stroke n, (%)	2 (1)	1 (0.7)	3 (0.9)	-
Bleeding n, (%)	4 (2.1)	2 (1.3)	6 (1.7)	-

COVID-19 infection was significantly common among patients using clopidogrel (p=0.010). There was no significant difference in mortality, bleeding, stent thrombosis, and stroke during hospitalization and at the one-year follow-up between patients with and without COVID-19 infection. Age, low ejection fraction, and high troponin values were associated with cardiac mortality in univariate and multivariate analyses (Table 4).

**Table 4:** Determination of risk factors for cardiac mortality during the one-year follow-up period HR, hazard ratio; CI, confidence interval

	Univariate		Multivariate	
	HR (95% CI)	p	HR (95% CI)	p
Age	1.11 (1.063-1.158)	<0.001	1.065 (1.092-1.143)	0.033
Gender (female)	2.307 (0.853-6.24)	0.100	2.386 (0.523-10.884)	0.262
Hypertension	0.966 (0.357-2.611)	0.945	0.663 (0.189-2.318)	0.519
Diabetes mellitus	0.88 (0.31-2.498)	0.810	2.075 (0.59-7.302)	0.256
Chronic kidney disease	1.888 (0.665-5.358)	0.233	0.531 (0.098-2.883)	0.463
Smoking	0.224 (0.073-0.687)	0.009	0.443 (0.114-1.716)	0.239
COVID history	1.872 (0.713-4.918)	0.203	1.822 (0.597-5.567)	0.292
Hemoglobin	0.807 (0.67-0.973)	0.024	1.048 (0.76-1.444)	0.777
Glomerular filtration rate	0.969 (0.95-0.988)	0.002	0.979 (0.937-1.022)	0.332
Troponin	1 (1-1.001)	<0.001	1 (1-1.001)	0.016
Low-density lipoprotein (mg/dL)	0.991 (0.978-1.004)	0.159	0.974 (0.903-1.05)	0.488
Ejection fraction	0.894 (0.843-0.947)	<0.001	0.913 (0.853-0.977)	0.008
Valve disease	5.94 (2.092-16.864)	0.001	3.26 (0.986-10.774)	0.053

The groups showed a significant difference in mean survival times for death from cardiac causes. The average survival time for the ticagrelor group was 11.1 months, 8.8 months for the clopidogrel group, and 11.8 months for the prasugrel group (p<0.001).

## Discussion

This study compared the efficacy and safety of P2Y12 inhibitors in patients presenting with STEMI during the COVID-19 era. There was no significant difference in clinical outcomes during hospitalization and at one-year follow-up in patients who had COVID-19 compared to those who did not. Regardless of COVID-19 history, cardiac mortality was significantly higher in the clopidogrel group compared to other groups.

COVID-19 has been shown to increase cardiovascular mortality due to its direct and indirect effects. The resulting prothrombotic environment, inflammatory pathophysiologic mechanisms that trigger plaque rupture, and local micro-thromboembolism can potentially impair perfusion and increase the risk of thrombosis in COVID-19 patients [1,10]. Several studies have indicated that patients with COVID-19 who are hospitalized with STEMI have a higher risk of both in-hospital and out-of-hospital mortality. Numerous investigations carried out on patients with STEMI during the COVID-19 pandemic have found that the diagnosis of STEMI in these patients was delayed, resulting in ineffective implementation of reperfusion therapies, and these patients have been linked to higher death rates. In-hospital mortality rates are between 20% and 40% in various studies [3]. In our study, in-hospital mortality was 11.1%, annual mortality was around 5%, and there was no difference in mortality between patients with and without COVID-19. In the recent International Study on Acute Coronary Syndrome (ISACS)-STEMI COVID-19 study conducted by De Luca et al., patients who underwent percutaneous coronary procedures between March 1 and April 30 in 2019 and 2020 were retrospectively reviewed. The study included 372 patients, and each COVID-19-positive patient was matched separately for age, gender, and hospital/geographic region with five COVID-19-negative patients. The study endpoints were in-hospital mortality, stent thrombosis, and heart failure. The usage of glycoprotein IIb-IIIa inhibitors and thrombectomy was more frequent in individuals who tested positive for COVID-19. There was also significantly higher in-hospital mortality, in-hospital definite stent thrombosis, and heart failure, with no difference in major bleeding complications in patients with COVID-19. It was also noted that there was no COVID-19-related pulmonary or infectious cause of increased mortality [11].

There could be several factors contributing to the variation in mortality, including but not limited to the following: our hospital maintained the same approach to primary revascularization procedures for patients with STEMI during the COVID-19 pandemic as we did prior to the pandemic. Even though guidelines suggest that thrombolytic therapy may be given to patients with active infections during the COVID-19 pandemic, our hospital promptly provided revascularization for patients with STEMI without delay. Delayed revascularization and late presentation of patients have been identified as contributing factors in multiple studies that have reported higher mortality rates.

Yuhang et al. conducted a meta-analysis of 38 studies including 79,753 patients, which revealed that patients with STEMI who were admitted to the hospital experienced a significant increase in the duration from symptom onset to their first medical consultation after the onset of the COVID-19 pandemic. Additionally, in-hospital mortality was found to be higher during the COVID-19 era compared to pre-COVID-19 era [12,13].

Increased risk of stent thrombosis due to COVID-19 has been demonstrated in several case series and studies [14-16]. COVID-19 was associated with a five-fold higher risk of definite in-hospital stent thrombosis independent of other factors. This observation could be a consequence of greater thrombus burden and there may be specific mechanisms involved in plaque rupture that may lead to an increased risk of thrombus formation and thrombotic complications in COVID-19 patients. Choudry et al. reported increased coronary thrombus burden, multiple thrombosed lesions, higher use of GP2b3a inhibitors, and thrombus aspiration in COVID-19 patients presenting with STEMI [7]. The mechanism of the relatively increased thrombus burden in COVID-19 patients presenting with STEMI remains unclear. Increased arterial thrombosis compared to increased venous thrombosis is attributed to increased platelet function and endothelial dysfunction. The systemic inflammatory response caused by COVID-19 infection has been shown to activate platelets and the coagulation cascade [17,18]. In our study, acute stent thrombosis was observed during hospitalization in three patients with COVID-19 and one patient without COVID-19. The differences in stent thrombosis rates were not statistically significant. This may be due to the fact that only a minority of patients present with STEMI during active COVID-19 infection. In addition, the severity of COVID-19 disease is closely related to the inflammatory response, and a limited number of our patients had severe COVID-19.

There was no notable variation in bleeding complications among patients with COVID-19 who received different antiplatelet medications in our study. Indeed, it is known that there is an increase in bleeding complications due to COVID-19-induced endothelial dysfunction. Routine use of P2Y12 inhibitors following STEMI can have varying effects. Ticagrelor and prasugrel are more potent antiaggregants than clopidogrel. Recent guidelines have recommended the primary use of ticagrelor and prasugrel in patients with STEMI. In the Intracoronary Stenting and Antithrombotic Regimen: Rapid Early Action for Coronary Treatment (ISAR REACT) study, it was demonstrated that prasugrel was superior to ticagrelor in reducing mortality. Nevertheless, for patients who are at a significantly high risk of bleeding, it is suggested that these medications be utilized for a limited duration and de-escalation treatments can also be employed. In our study, the rate of clopidogrel use was approximately two times higher than that of ticagrelor and prasugrel. Again, in the subgroup of patients who had COVID-19, the rate of clopidogrel use was almost two-fold higher. Therefore, bleeding complications were considerably low when compared to previous studies. The reason for the low incidence of bleeding could be attributed to the fact that the study population consisted of relatively low-risk patients, taking into account their age and other risk factors. In a recent study, patients with STEMI were compared in the pre-COVID-19 era and COVID-19 era, and bleeding rates were similar in both groups. Similarly, the rate of clopidogrel use was higher than that of ticagrelor (60-40%), and there were no patients receiving prasugrel [19]. In a study comparing patients with STEMI admitted pre-COVID-19 era and COVID-19 era, although ticagrelor was predominantly used, there was no difference in in-hospital mortality and bleeding outcomes, similar to our results [20].

During the pandemic, Kiriş et al. reported a delay in the admission time and a reduction in the hospitalization time of 1788 Turkish patients with STEMI. In the same study, mortality, cardiogenic shock, and thrombus burden were higher in patients with COVID-19 infection compared to those without. Similar to our study, the majority of patients were treated with clopidogrel, and no difference was observed in the bleeding endpoint [21].

Some limitations of the study are as follows: 1) retrospective design: the study has a retrospective design, so the data was collected retrospectively. A prospective study could provide stronger evidence; 2) sample size: although the study included 390 patients, having a larger sample size could make the results more reliable and generalizable; 3) single-center study: the study was conducted at a single center, and the results may vary in patients from other centers; 4) COVID-19 and STEMI relationship: the study focuses on the relationship between COVID-19 infection and STEMI, but more comprehensive research is needed to fully understand the impact of COVID-19 on other clinical outcomes in STEMI patients; 5) drug selection: the choice of P2Y12 inhibitors was based on the patient's clinical condition and comorbidities. Conducting randomized controlled trials would be beneficial to assess the impact of these drug choices on outcomes more accurately. Additionally, patients using clopidogrel were at clinically higher risk than the other groups, which could potentially affect the results; 6) follow-up period: the follow-up period in the study was set as one year, and a longer follow-up could provide more definitive results; 7) impact of other variables: other potential factors not mentioned in the study might have an effect on the outcomes.

## Conclusions

 This study showed that there was no significant difference in mortality, bleeding, stroke, and thrombotic complications among patients treated with different P2Y12 inhibitors, regardless of the presence or absence of COVID-19 infection. The study concluded that clopidogrel was associated with increased cardiovascular mortality compared to ticagrelor and prasugrel. However, there were no significant differences in bleeding complications among patients receiving different P2Y12 inhibitors. The findings of this study suggest that it would be appropriate to continue applying antithrombotic therapy as recommended by the guidelines in patients with STEMI during the COVID-19 era.
